# Accuracy to detection timing for assisting repetitive facilitation exercise system using MRCP and SVM

**DOI:** 10.1186/s40638-017-0071-5

**Published:** 2017-11-07

**Authors:** Satoshi Miura, Junichi Takazawa, Yo Kobayashi, Masakatsu G. Fujie

**Affiliations:** 1 Faculty of Science and Engineering, Waseda University, 3-4-1, Okubo, Shinjuku-ku, 169-8555 Tokyo, Japan; 2 Graduate School of Science and Engineering, Waseda University, 3-4-1, Okubo, Shinjuku-ku, 169-8555 Tokyo, Japan; 3 Healthcare Robotics Institute, Future Robotics Organization, Waseda University, 3-4-1, Okubo, Shinjuku-ku, 169-8555 Tokyo, Japan

**Keywords:** Neurorehabilitation, Repetitive facilitation exercise, Brain–machine interface, Motor command detection

## Abstract

This paper presents a feasibility study of a brain–machine interface system to assist repetitive facilitation exercise. Repetitive facilitation exercise is an effective rehabilitation method for patients with hemiplegia. In repetitive facilitation exercise, a therapist stimulates the paralyzed part of the patient while motor commands run along the nerve pathway. However, successful repetitive facilitation exercise is difficult to achieve and even a skilled practitioner cannot detect when a motor command occurs in patient’s brain. We proposed a brain–machine interface system for automatically detecting motor commands and stimulating the paralyzed part of a patient. To determine motor commands from patient electroencephalogram (EEG) data, we measured the movement-related cortical potential (MRCP) and constructed a support vector machine system. In this paper, we validated the prediction timing of the system at the highest accuracy by the system using EEG and MRCP. In the experiments, we measured the EEG when the participant bent their elbow when prompted to do so. We analyzed the EEG data using a cross-validation method. We found that the average accuracy was 72.9% and the highest at the prediction timing 280 ms. We conclude that 280 ms is the most suitable to predict the judgment that a patient intends to exercise or not.

## Background

The number of cerebral stroke patients is increasing worldwide. For example, in Japan, cerebral stroke patients exceeded 2.8 million people in 2015 [1]. Patients often suffer from aftereffects following a stroke, the most frequent of which is hemiplegia. To recover motor function following hemiplegia, patients must endure a long course of difficult rehabilitation. Many studies have investigated methods to shorten the recovery time through efficient rehabilitation after hemiplegia [2, 3].

Neurorehabilitation has been shown to be very efficient [4, 5]. Neurorehabilitation is a method to prompt the recovery of the injured neural system. Repetitive facilitation exercise is drawing attention as a particularly effective rehabilitation. Kawahira demonstrated the efficacy of repetitive facilitation exercise [6]. Patients using repetitive facilitation exercise can recover motor control three times faster than those using usual therapy [7]. Repetitive facilitation exercise also improves the paralysis part to health compared with usual therapy [8].

**Fig. 1:**
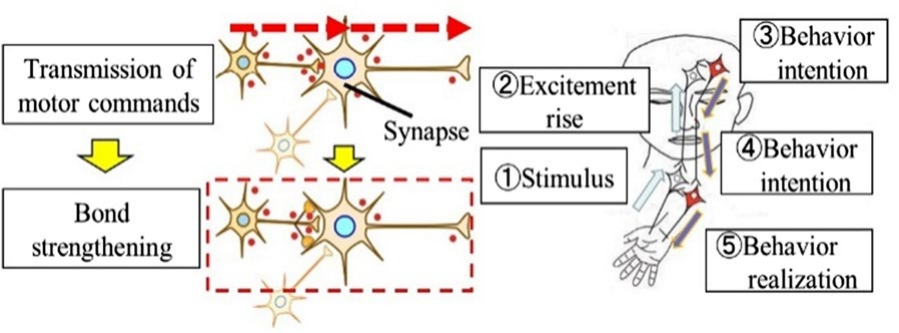
Principle of repetitive facilitation exercise. When a motor command occurs in the brain, the synapse bond strengthens. The neural networks are excited. At this time, the therapist stimulates the patient’s paralyzed arm. This stimulation is transmitted to the brain. When the intention to move the arm and the stimulus overlaps, the brain recognizes this as if the patient had moved the paralyzed hand. This recognition promotes the reconstruction of the nerve pathway to the paralyzed arm

Figure 1 shows the mechanism of the repetitive facilitation exercise. The patient imagines moving a paralyzed body part. Within the patient’s body, motor commands travel from the brain, through the spinal cord, to the paralyzed part. The therapist then stretches the paralyzed part of the patient using physical or electrical stimulation before the motor command reaches the spinal cord. This stimulation excites the nerves in the spinal cord and activates the path of the motor command. Because the motor command can pass more easily through the nerve pathway, the patient becomes likely to regain the ability to move the paralyzed part unaided.

The most important point of repetitive facilitation exercise is the stimulation timing. It is necessary for the stimulation to occur before the motor command reaches the spinal cord for success of the repetitive facilitation exercise. However, it is difficult to successfully perform repetitive facilitation exercise because even a skilled therapist cannot detect the timing of the motor command. The success of repetitive facilitation exercise is dependent on the intuition and experience of the therapist.

Even an experienced therapist cannot achieve a 100% success rate with the repetitive facilitation exercise because therapists cannot detect patient motor intentions. It has been reported that five of twelve patients showed increased motor evoked potential after repetitive facilitation exercise by therapists [21]. This shows that the nervous systems of these patients underwent some reconstruction. Although there are individual differences, it is said that the conventional success rate is about five of twelve, that is just 45%. To improve the higher success rate than the target value 45%, therapists require a system to assist repetitive facilitation exercise. An upper limb reaching device has been proposed to reduce fatigue and pain in the paralyzed arm and to decrease the burden on the therapist [9]. The patient repeats inward and outward movements to push the front and back buttons alternately. When the patient pushes the button, the device generates vibrations and electric stimulation to make it easier to move the paralyzed arm. However, the device cannot control the accurate stimulation timing because the device cannot detect the motor command generated in the brain.

### Related work

There have been many studies of real time detecting the motor command in brain. Most of these have used electroencephalogram (EEG) because it has a higher time resolution than other brain measurement devices. EEG is simple to analyze in real time. For example, Lucian reported that EEG can show the steering timing of a driver during driving [10, 11, 12]. He measured and analyzed EEG data while participants operated a driving simulator. Using this method, the turn direction was detected 811 ms before steering with an accuracy of 74.6%. In another study, Choi showed that a brain–machine interface system using EEG could be used to control a wheelchair [13]. This system analyzed EEG data and moved forward or turned left or right based on the measured EEG signals within 125 ms.

These studies are useful for realizing real-time brain–machine interfaces for healthy individuals. However, these studies have not been adapted to the rehabilitation.

### Objective

**Fig. 2:**
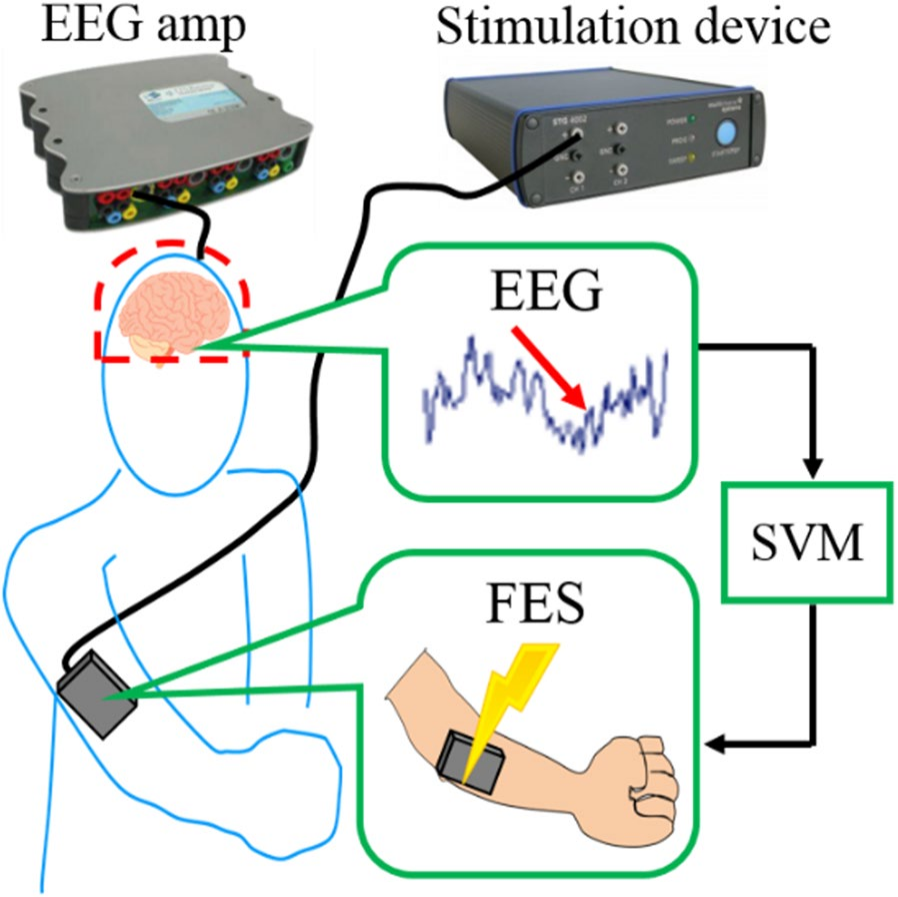
Proposed system. The system measures the patient’s EEG using the EEG amp and analyzes EEG data using the SVM. The system actuates FES using the stimulation device. The system stimulates the paralyzed part electrically

**Fig. 3:**
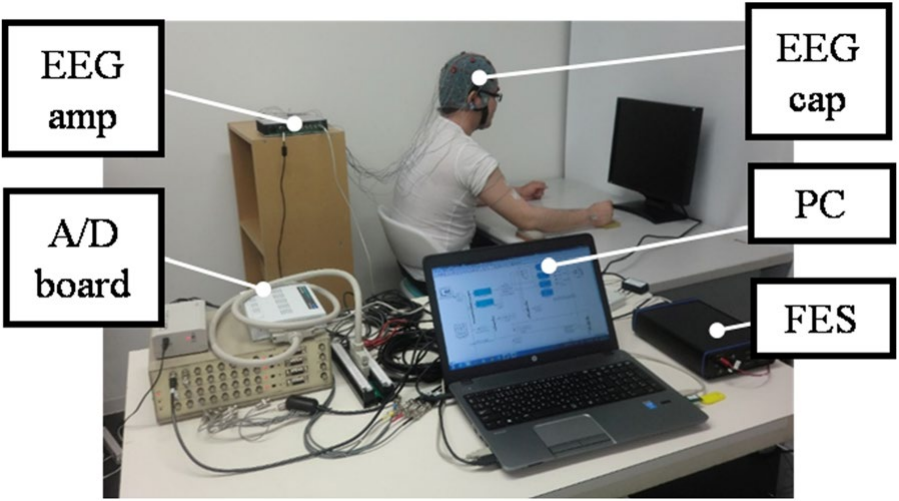
Overview of the system. An EEG cap is set on the patient’s head. EEG data are measured by the EEG cap, amplified by the EEG amp and output via the A/D board to a PC. The PC is used to analyze the EEG data and actuate the FES

**Fig. 4:**
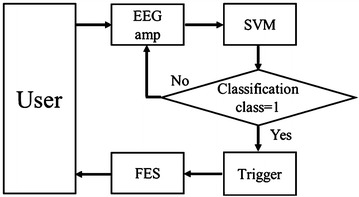
Flow of the system. The system measures the patient’s EEG data and analyzes it using SVM. If classification class by SVM is 1, the trigger is on and the system actuates the FES to stimulate the patient

Our motivation is to develop a brain–machine interface system to assist repetitive facilitation exercise. The system detects motor intention in real time by measuring the brain and stimulates the paralyzed body part before the motor command reaches the spinal cord, as shown in Fig. 2. The system overview and flow are shown in Figs. 3 and 4. The system is consisted of the EEG measurement device and FES generator, shown in Fig. 3. The system detects motor commands from the patient’s EEG data using support vector machine (SVM). It then stimulates the paralyzed part by functional electric stimulation (FES) before the motor command reaches the spinal cord. The system provides stimulation at the same time the SVM detects the motor command; thus, the stimulation occurs after the motor command is produced, but before it reaches the spine.

**Fig. 5:**
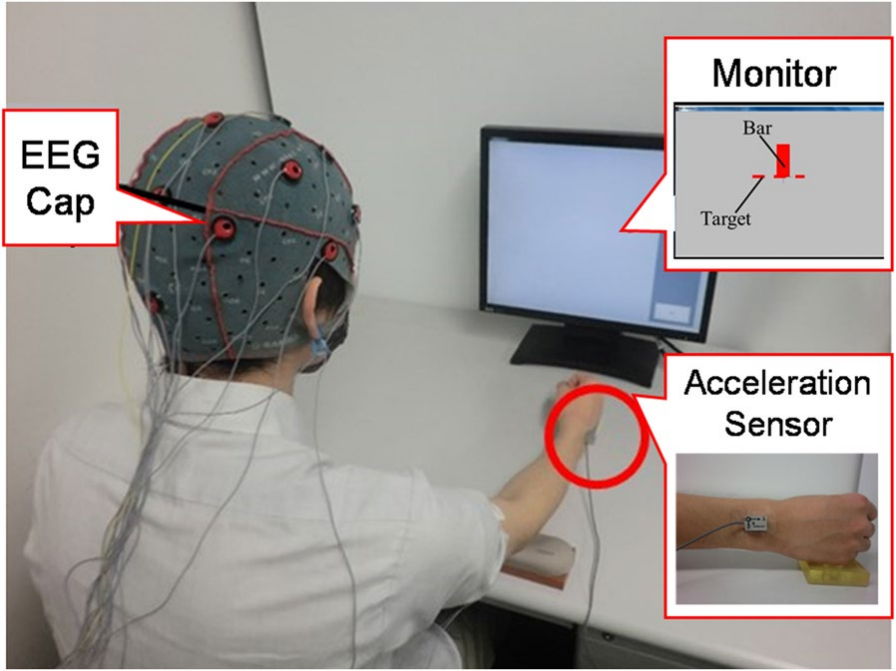
Experimental overview. The EEG cap is set on the participant’s head, and the accelerometer is put on the participant’s wrist. First, the participant remains relaxed. A red bar is displayed on the monitor and shortens gradually. At the same time the bar disappears, the participant bends their elbow. We detect when the participant bends the elbow by analyzing the acceleration of the wrist

In this paper, we validate our proposed motor command detecting method. The prediction accuracy changes as to the timing before action. It is not clear that the accuracy changes as to the prediction timing changing. We clarify the appropriate prediction timing by the accuracy of the system using SVM. We constructed an SVM system and carried out experiments to clarify the detection ratio of motor commands. In the experiment, we collected EEG data when the participant bent their elbow, shown in Fig. 5. To facilitate the timing, we displayed a bar on a monitor to indicate to the participant when to bend their elbow. The bar was displayed on the monitor and shortened gradually until it disappeared. At the same time the bar disappeared, the participant had to bend their elbow. We analyzed EEG data using a cross-validation method to clarify the detection ratio of the motor command. We confirmed that the calculated detection ratio was above the target value, verifying the utility of the proposed system.

## Methods

### Participants

Three healthy participants (male, age 22–23, two right-handed and one left-handed) were enrolled in the experiment. We did not enroll paralyzed patients because the aim of the present study was only to validate the proposed method for detecting motor commands. Informed consent was obtained from all participants. All participants attested to having slept well the night before the experiment to exclude the influence of sleep deprivation. All participants did not intake the drugs such as caffeine, alcohol, nicotine, and other medicines. The experiments were approved by the Waseda University Institutional Review Board (No. 2014-156).

### Experimental setup

**Fig. 6:**
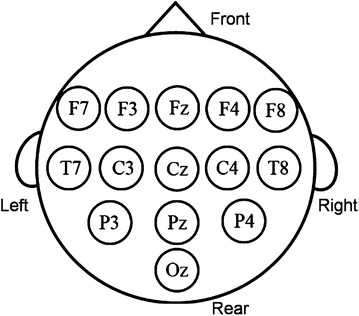
Measured points on the EEG cap, viewed from directly above the patient’s head. The 14-ch EEG data are measured at these points

We used an EEG (g. USBamp, gtec, USA) to measure brain activity. We set up the device as shown in Table 1. The sampling frequency was set to 256 Hz because the real-time measurement using MRCP needs a high temporal resolution. We used 17 analog input channels and 1 GND passive channel. On an EEG cap, 14 channels were located to measure EEG based on the international 10–20 system, as shown in Fig. 6. The reference electrode was put on the earlobe.

**Table 1:** Device settings of a g. USBamp and ACL300

Title	Specification	Mounting position
Sampling rate Hz	256	
Analog input channel ch	17	
Mounting position	ch1	F7
	ch2	F3
	ch3	Fz
	ch4	F4
	ch5	F8
	ch6	T7
	ch7	C3
	ch8	Cz
	ch9	C4
	ch10	T8
	ch11	P3
	ch12	Pz
	ch13	P4
	ch14	Oz
	ch15	Acceleration sensor ch1 (X-axis)
	ch16	Acceleration sensor ch2 (Y-axis)
	ch17	Acceleration sensor ch3 (Z-axis)
	GND	Nz

**Fig. 7:**
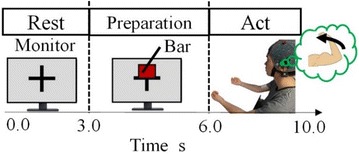
One measurement session. The rest period is 0–3 s from starting measurement. The preparation period is 3–6 s and includes the appearance, gradual shortening and disappearance (at 6 s) of the red bar. The act period is 6–10 s, beginning at the same time as the bar disappears. In the act period, the participant bends their elbow

We used a three-degree-of-freedom accelerometer (ACL300, Biometric Inc., USA) to detect the time when the participant moved their arm. We affixed the accelerometer to the participant’s wrist as shown in Fig. 6 and connected it to an analog input–output board (AIO-163202FX-USB, Contec Inc., USA) to get the analog input value. The accelerometer used three channels, as shown in Table 1.

### Experimental task

We conducted the experiment in a closed room to minimize noise disturbances. During the experiment, the participant did not talk and sat still. In addition, we asked the participant to try to avoid swallowing saliva or blinking hard. The temperature was 20 ± 15 °C, and the humidity was 45–85%.

**Fig. 8:**
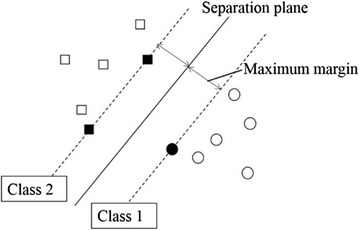
Classification by the support vector machine (SVM). The separation plane divides the data into Class 1 and Class 2. The margin is the distance between the separation plane and the closest data point. SVM sets the separation plane to maximize the margin

We put the EEG cap and the accelerometer on the participant. The experimental procedure is shown in Fig. 8. In the initial state, the participant relaxed with their right forearm resting on the desk, palm up. One measurement session consisted of rest, preparation and act periods within 10 s. During the rest period, from 0 to 3 s, the participant relaxed and looked at the monitor. During the preparation period, from 3 to 6 s, a red bar was displayed on the monitor and became smaller until disappearing at 6 s, as shown in Fig. 7. During the act period, after 6 s, at the same time as the red bar disappeared, the participant bent their arm at the elbow. The participant kept their elbow bent for approximately 0.5 s and then set their arm to the initial resting state. This experimental procedure was performed 100 times by each participant.

### Experimental condition

The experimental condition is the prediction timing. The prediction accuracy changes as to the timing before action. However, the timing is influenced by the recognition delay. Even the participant bent his/her elbow as soon as the disappearance of the bar, and there is actually the delay because it takes time to recognize it. The recognition delay is said to about 200 ms, but there is no clarity of accurate delay. In this paper, the experimental condition is the prediction timing around 200 ms before action timing. The condition is 70, 140, 210, 280 and 350 ms before action timing.

### Analysis

#### Movement-related cortical potential (MRCP)

We focused on the functions of EEG. For example, event-related potential is the electric fluctuation detected from neurons following light or sound stimulus [14]. The P300 speller, a communication device for severely paralyzed patients, utilizes the event-related potential function. Event-related desynchronization is another function in which the power spectrum of the EEG alpha band decreases following motor commands [15]. Event-related desynchronization is often used in rehabilitation systems. In the present study, we used movement-related cortical potential (MRCP). MRCP is the change in EEG signal resulting from the plan and action of voluntary exercise [16, 17]. MRCP is detectable before and after exercise. In particular, MRCP that starts about 800 ms before exercise is called the motor readiness potential. We hypothesized that the motor readiness potential would show the timing of when a motor command occurs in the brain.

#### Support vector machine (SVM)

To detect MRCP, a pattern identification unit is required. There are two kinds of pattern identification unit: One uses a parametric method for which the probability distribution of data is known in advance and another uses a nonparametric method which requires collected data because the probability distribution of data is unknown. We used the nonparametric method because EEG data are different for each patient.

We employed a support vector machine (SVM) because it can divide known data into two classes [18, 19, 20]. Compared with other algorithms, SVM is suitable to judge the two classes that the human tends to move his body or not. Using this SVM system, we divided the EEG data into data during rest and data during action. The system needs to detect EEG data during action as a motor command.

SVM is a supervised learning method that can construct pattern identification to two classes. SVM learns the parameters required to maximize the margin from training sample data. SVM decided the two outputs using the following:

1 $$ y = sign\left( {W^{T} x - h} \right) $$

where *W* is the weight parameter and *h* is the threshold. If *u* > *0, sign(u)* is 1. If u≤0, *sign(u)* is − 1. Figure 7 shows the SVM classification. The Class 1 and Class 2 mean that the label of each class is 1 and -1. The margin is the distance between the separation plane and the closest data point. SVM finds an optimal value of *W* to maximize the margin.

#### Judgment of movement from acceleration

We recorded the time when the participant bent their elbow using the accelerometer. We calculated the following:

2 $$ V_{xyz} = \sqrt {V_{x}^{2} + V_{y}^{2} + V_{z}^{2} } - 0.1 $$

where *V*_*xyz*_ is the combined acceleration, and *V*_*x*_ *, V*_*y*_ and *V*_*z*_ are the X-, Y- and Z-axis components of the acceleration. We clarified the maximum value of the acceleration during the rest period. We set the maximum acceleration value during rest period as the threshold of the movement starting judge. We judged when the acceleration was over the threshold as the timing when the participant bent the elbow.

To detect motor commands, we used EEG data from 0 to 2 s during each measurement session as the feature quantity during rest. EEG data at 210 ms after the acceleration of the wrist were considered the threshold for motor command. There are two reasons to set this threshold to 210 ms: One is the human cognitive delay. In this experiment, the participant bent and their elbow based on a signaling displayed on a monitor—the disappearance of the red bar. Therefore, we considered that it would take 200 ms after the bar disappears for the participant to recognize the bar disappearance. Another reason is machine delay. There is a machine delay of 10 ms from motion intention detection by the SVM to actuation of the FES. From the above, we set the expected delay to 210 ms.

**Fig. 9:**
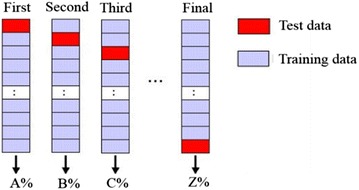
Cross-validation method. The red blocks represent test data. The purple blocks represent training data. First, we considered the first data block as test data, and all other data blocks training data. Second, we considered the second data block as test data, and all other data blocks training data. We repeated this procedure *k* times

We clarified the identification ratio using cross-validation of the data from 100 trials by each participant. Figure 9 shows the cross-validation method. We divided the original data into *k* blocks. Using the first block as test data and the other data as training data, we calculated the discrimination ratio. Next, using the second data as test data and the other data as training data, we calculated the discrimination ratio again. By repeating the above procedure *k* times, we used the average of *k* values for discrimination rate to estimate the accuracy of the model.

**Fig. 10:**
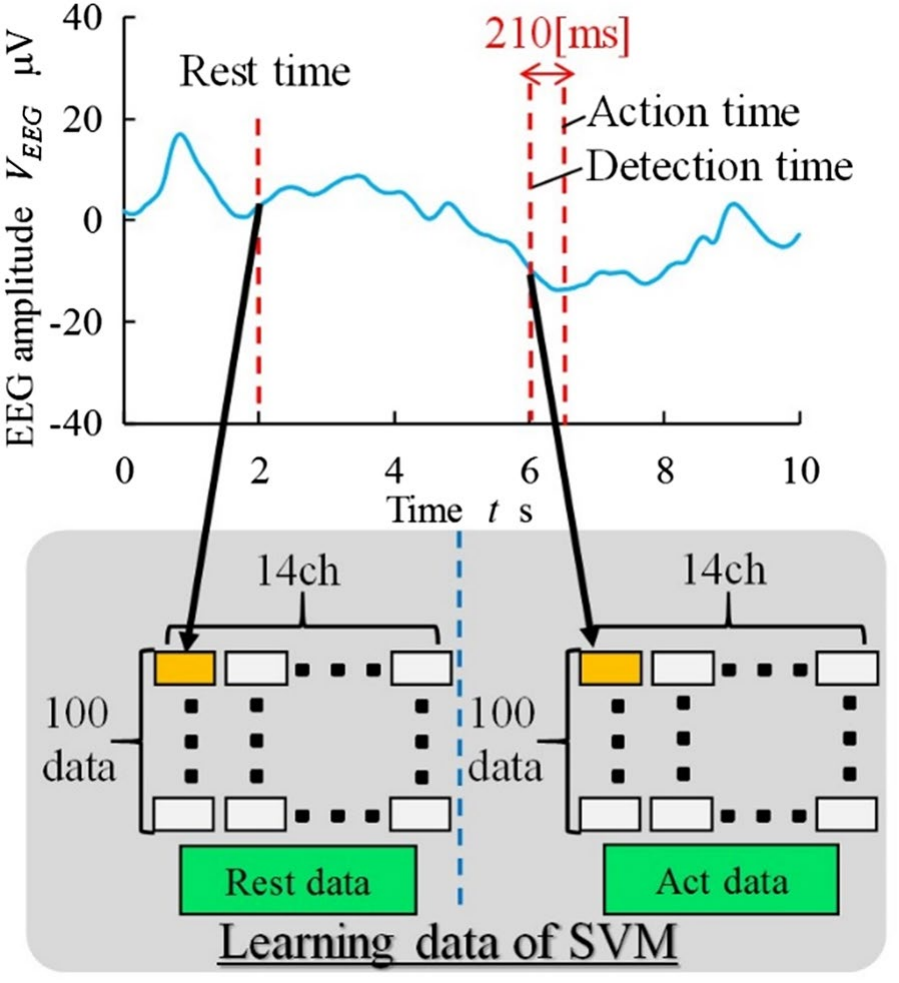
Construction method of the SVM. The graph above is a sample of EEG data. SVM learns the data during rest and act periods

Figure 10 shows the procedure for constructing the SVM. The action time is the timing when the acceleration of the wrist was over the maximum during rest. The detection time is 210 ms before the action time. We selected a value for *k* of 10, and the feature quantity was all 14-ch EEG data during 210 ms from the detection time to the action time.

To compare with other detection timings, we validate the discrimination rate by each 70 ms via cross-validation. The condition is 70, 140, 210, 280, 350 ms.

## Results and discussion

Discrimination ratio by each detection timing is shown in Table 2. This discrimination ratio is about 70%. The results show that SVM could detect the MRCP effectively. Particularly, the highest average is 72.9% at 280 ms so we determine that the most appropriate detection timing is 280 ms.

**Table 2:** Experimental result of detection rate

Subject name	Detection rate for shift time *r* %				
	70 ms	140 ms	210 ms	280 ms	350 ms
Average all subjects	70.9	72.0	71.8	72.9	72.5
Subject A	69.0	68.0	69.0	67.5	73.5
Subject B	65.6	69.9	69.4	69.4	65.1
Subject C	78.2	78.2	77.1	81.9	79.3

**Fig. 11:**
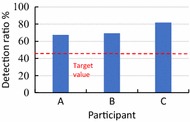
Detection rate for each subject. The discrimination ratios of participants A, B and C are 69, 69.4 and 77.1%, respectively. The target value is 45%

We clarified the discrimination ratio at 280 ms for each participant, as shown in Fig. 11. The discrimination ratios of participants A, B and C were 67.5, 69.4 and 81.9%, respectively. The average was 72.9%; this is over the 45% target value.

Using EEG data 210 ms after the red bar disappeared, all discrimination data were at least 67%. This result was above the 45% target value. This indicates that using EEG data sorted by SVM, the proposed system can perform FES on paralyzed patients with adjustable timing. Repetitive facilitation exercise administered using the proposed EEG system is potentially more successful than that administered by a therapist.

In the present study, we carried out the experiment by only three participants. We should conduct experiments using more participants. In addition, the EEG data were collected from only healthy subjects. For some stroke patients, although the neural system is different from the healthy subject, the sensory recognition motor loops would be same as to healthy because the neural system cannot feedback but can feedforward. We should validate the detection using EEG signals of paralyzed patients compared with the healthy people. In future work, we will develop the system using FES.

## Conclusions

In the present study, we proposed a brain–machine interface system to assist repetitive facilitation exercise. As a result, the average accuracy was 72.9% and the highest at the prediction time 280 ms. We conclude that 280 ms is the most suitable to predict the judgment that a patient intends to exercise or not. In future work, we will develop this repetitive facilitation exercise assistance system.
